# Dehydrocorydaline maintains the vascular smooth muscle cell contractile phenotype by upregulating Spta1

**DOI:** 10.1038/s41401-024-01464-9

**Published:** 2025-01-20

**Authors:** Yuan-ye Dang, Cui Chen, Qiu-fen Wei, Li-feng Gao, Shun-chi Zhang, Yong-xian Li, Xiao-yan Dai

**Affiliations:** 1 The Fifth Affiliated Hospital, Guangdong Province & NMPA & State Key Laboratory, School of Pharmaceutical Sciences, Guangzhou Medical University, Guangzhou, 511436 China; 2 Clinical Laboratory, Maternal and Child Health Hospital, Taiyuan, 030012 China; 3 Department of Clinical Laboratory, Guangdong Provincial Hospital of Integrated Traditional Chinese and Western Medicine, Foshan, 528253 China; 4 Clinical Research Institute, the Second Affiliated Hospital, Hengyang Medical School, University of South China, Hengyang, 421002 China

**Keywords:** atherosclerosis, dehydrocorydaline, vascular smooth muscle cell, phenotypic switching

## Abstract

Vascular smooth muscle cell (VSMC) phenotypic switching plays a crucial role in the initiation and progression of atherosclerosis. Dehydrocorydaline (DHC), a major active component of the traditional Chinese herbal medicine *Rhizoma Corydalis*, exhibits diverse pharmacological effects. However, its impact on VSMCs remains largely unknown. This study aims to investigate the effects and underlying mechanisms of DHC in phenotypic switching of VSMCs. Our study revealed that DHC increased the mRNA and protein levels of rat VSMC contractile phenotype markers, such as calponin 1 (Cnn1), myosin heavy chain (Myh11, SM-MHC), smooth muscle 22α (Sm22α), and alpha-smooth muscle actin (Acta2, α-SMA) in a time- and dose-dependent manner. Additionally, DHC inhibited platelet-derived growth factor-BB-induced VSMC proliferation and migration. In *Apoe*^*−/−*^ mice, DHC treatment resulted in reduced carotid plaque areas and macrophage infiltration, along with increased contractile phenotype marker expression. RNA sequencing analysis revealed a significant upregulation of spectrin alpha, erythrocytic 1 (Spta1) in DHC-treated rat VSMCs. Strikingly, Spta1 knockdown effectively negated the increase in contractile phenotype marker expression in VSMCs that was initially prompted by DHC. Therefore, DHC preserves the VSMC contractile phenotype through Spta1, thereby attenuating carotid artery atherosclerotic plaques in *Apoe*^*−/−*^ mice. This study provides evidence supporting the potential use of Chinese herbal medicines, particularly those containing DHC such as *Rhizoma Corydalis*, in the treatment of atherosclerotic cardiovascular disease, thus expanding the clinical application of such herbal remedies.

## Introduction

In recent years, global cardiovascular morbidity and mortality have continued to rise, with cardiovascular disease (CVD) deaths constituting approximately one-third of all global fatalities. The leading causes of CVD deaths, accounting for over 95%, include ischemic heart disease, stroke, hypertensive heart disease (eventually leading to heart failure), cardiomyopathy, rheumatic heart disease, and atrial fibrillation [1]. Atherosclerosis, characterized by long-term accumulation and transformation of lipids, inflammatory cells, and smooth muscle cells, as well as necrotic cell debris in the intimal space, is the most common pathogenesis of CVD. Current treatment modalities predominantly involve lifestyle improvements, drug therapy, and arterial blood flow reconstruction through percutaneous approaches or surgery. Statins, which are recognized for their outstanding lipid-lowering effects, serve as first-line drugs for atherosclerosis treatment. However, their capacity to reduce CVD incidence by only 40%–50%, coupled with a high recurrence rate of cardiovascular events in patients with residual inflammation risk, underscores the pressing need for novel therapeutic drugs [2]. Recent trials, such as CANTOS and COLCOT, have indicated that targeting inflammation can complement CVD treatment; however, this approach is associated with a higher incidence of infection-related adverse events in individuals undergoing anti-inflammation therapy [3, 4]. Therefore, the search for new effective therapeutic drugs is paramount.

Vascular smooth muscle cells (VSMCs), which reside in the media of the vascular wall, play a central role in atherosclerosis. While mature VSMCs exhibit a contractile phenotype, they can transition to a synthetic phenotype when stimulated [5]. This phenotypic switch involves the downregulation of contractile VSMC-specific markers, including alpha-smooth muscle actin (α-SMA, Acta2), smooth muscle myosin heavy chain (SM-MHC, Myh11), Calponin 1 (Cnn1), and smooth muscle 22α (Sm22α), resulting in increased proliferation and migration abilities [6–8].

The significance of VSMC phenotypic switching in atherosclerosis is well documented, and inhibition of this process is beneficial in advanced atherosclerosis. The regulation module of myocardin-serum response factor is the core component of phenotypic regulation. Studies by Amarnath Talasila revealed that re-expression of myocardin increases VSMC contractile phenotype markers in murine carotid arteries, with decreased smooth muscle cell migration and proliferation [9]. Ye Ding et al. demonstrated that AMPKα2 deletion induced VSMC phenotypic switching and promoted features of atherosclerotic plaque instability in an NF-κB-KLF4-dependent manner [10].

Dehydrocorydaline (DHC) is an alkaloid extracted and isolated from the tubers of *Rhizoma Corydalis*, a traditional Chinese herb. It has been shown to have anti-inflammatory [11–13], anti-allergic [14], anti-tumor [15–17], and norepinephrine release inhibiting effects [18]. Our published study indicated that DHC improved atherosclerosis and aortic compliance in Western diet-fed *Apoe*^*−/−*^ mice by ameliorating systemic and vascular inflammation [19]. However, the role of DHC in VSMCs remains unexplored, and whether DHC affects atherosclerotic plaques by regulating VSMC phenotypic switching is unknown. This study aims to explore the role and mechanism of DHC in VSMC phenotypic switching and plaques using a mouse model of carotid artery injury and in vitro cell experiments.

## Materials and methods

### Chemicals and reagents

DHC (30045-16-0, Chengdu, China) was purchased from Chroma-Biotechnology Co., Ltd., and dissolved in dimethyl sulfoxide (DMSO, Sigma-Aldrich). For intraperitoneal injections, DHC was prepared in a formulation of 1% DHC, 30% PEG30 (Selleck, S6704-100 g), 5% Tween 80 (Selleck, S6702-100 mL), and 64% water. Platelet-derived growth factor-BB (PDGF-BB) was used for cell treatment.

### Animals

Male *Apoe*^*−/−*^ mice, acquired from Beijing HFK Bioscience Co., Ltd., were housed under specific pathogen-free conditions at the Experimental Animal Center of Guangzhou Medical University. Mice were maintained on a 12 h light-dark cycle with *ad libitum* access to a standard diet and water for 12 weeks. The mice were randomly divided into vehicle and DHC groups. The carotid artery injury model followed Takeshi Sasaki’s method [20], involving ligation of the right common carotid artery (RCCA) and subsequent polyethylene cuff placement (427410; BD Biosciences, San Jose, CA, USA). Daily intraperitoneal injections of DMSO or DHC (5 mg/kg) were administered. The RCCA was then collected for histopathological analysis via frozen sectioning.

### Cell culture

Primary rat smooth muscle cells were isolated from the aortic tunica media of male Sprague-Dawley rats (150–180 g) according to the tissue block culture method [21]. Cells were cultured in Dulbecco’s Modified Eagle’s Medium (DMEM, C11995500BT, Gibco) supplemented with 10% fetal bovine serum (Gibco, 10091–148) + 1% penicillin/streptomycin (15140-122, Gibco) and maintained at 37 °C and 5% CO_2_, with an average of six to seven passages per experiment.

### RNA-sequencing (RNA-seq)

Following treatment with DMSO or DHC, rat primary VSMCs were harvested using Trizol for total RNA extraction. Transcriptome sequencing was carried out by Guangzhou IGE Biotechnology Co., Ltd., using the Illumina NovaSeq platform. Sequenced mRNA was aligned with the reference genome to quantify gene expression levels and identify differentially expressed genes. Functional annotation and enrichment analysis were performed to examine the biological implications of these genes.

### Western blotting

The cell treatment was stopped by washing the cells twice with ice-cold phosphate-buffered saline (PBS). To lyse the cells, radioimmunoprecipitation assay lysis buffer was applied for 10 min, followed by cell scraping using a cell scraper. The resulting lysate was then transferred to an EP tube and centrifuged at 13,000 × *g* for 10 min, allowing for the collection of the supernatant. The protein concentration of the supernatant was determined using a BCA Protein Assay Kit (P0009, Beyotime). Subsequently, 4× loading buffer was added to the supernatant and thoroughly mixed, and then the mixture was heated at 100 °C for 10 min to denature the proteins. Each well of an 8% polyacrylamide gel was loaded with 30 μg of protein and was flanked with pre-stained protein markers (abs923, Absin; mP102-02, Vazyme). Electrophoresis was performed at a constant voltage to separate the proteins, which were then transferred to a nitrocellulose membrane (66485, PALL) using a steady current. The membrane was blocked at room temperature using 5% skim milk for 1 h and subsequently incubated overnight with the following primary antibodies: SM-MHC (ab53219, Abcam; 21404-1-AP, Proteintech), Cnn1 (ABT129, Millipore), spectrin alpha, erythrocytic 1 (SPTA1) (abs134307, absin), and β-actin (sc-47778, Santa Cruz). After washing, the membrane was incubated with a horseradish peroxidase-conjugated secondary antibody for 1 h. Detection was carried out using a horseradish peroxidase chemiluminescent substrate (WBKLS0500, Millipore), and the blot was visualized and analyzed for density using an Amersham™ Imager 600 (GE, MA, USA). Semi-quantitative analysis of the results was performed using ImageJ software [22].

### Real-time quantitative PCR (RT-qPCR)

After the cell treatment was completed, the cells were washed twice with ice-cold PBS, and RNAex Pro RNA Reagent (21102, AG) was added to extract the total RNA of rat VSMCs. The RNA purity and concentration were determined using a Thermo Scientific NanoDrop One. cDNA was synthesized using a reverse transcription kit (11706, AG), and the mRNA level was detected with a SYBR Green Real-time qPCR Master Mix Kit (Q711-02, Vazyme). RT-PCR was performed on a LightCycler® 480 Instrument II (Roche Applied Sciences, CA, USA). The primers used are shown in Table 1.

**Table 1:** The primer sequences of target genes used in the experiments.

Rat primers	Forward (5′-3′)	Reverse (5′-3′)
*Cnn1*	GCCCAGAAATACGACCACCA	TGGAGCTTGTTGATAAATTCGCA
*Myh11*	ATCACGGGGGAGCTGGAAAA	AATGAACTTGCCAAAGCGGG
*Sm22α/Tagln*	TTCTGCCTCAACATGGCCAAC	CACCTTCACTGGCTTGGATC
*Acta2*	CATCCGACCTTGCTAACGGA	AGAGTCCAGCACAATACCAGT
*Spta1*	GCGAGAGGAACCAGTCAACA	GCGTTCTTCTGCCCGATCTA
*Gapdh*	ATTGTCAGCAATGCATCCTG	ATGGACTGTGGTCATGAGCC
*Opn/Spp1*	CAGTCGATGTCCCTGACGG	GTTGCTGTCCTGATCAGAGG
*Pcna*	CTGCTGGGACATCAGTTCGG	TGGACATGCTGGTGAGGTTC
*Klf4*	CTGAACAGCAGGGACTGTCA	GTGTGGGTGGCTGTTCTTTT
*Myocd*	CAAGGGTGTGCACAGATGAC	TAGGATGGGGGCTGGGTTAT
*Mlck*	CAGGCTCGAAGCCCTCTT	TGCTTGCTCCTTGTTCTCCTC
*Foxf1*	CCAGCAGAATTGCAAGGCATCC	TGGCCTCACCTCACATCACA
*Tet2*	GAGGAGCAGAAGGAAGCAAG	CACCGTAGCAGAACAGGAAC
*Gpx4*	AAGACGAAGGGGAAGACGCACATC	AAGACGAAGGGGAAGACGCACATC
*Ptgs2*	TCCTCCTGTGGCTGATGACT	CGGGATGAACTCTCTCCTCA
*Mmp2*	TGGACCCTGAGACAGTGGAT	GCTGCTGTATTCCCGACCATT
*Mmp9*	ACCGCCAACTATGACCAGGATAAGC	AAGACGAAGGGGAAGACGCACATC
*Cd68*	GTTCCCAGCCATGTGTTCAG	TCCAAAGGTAAGCTGTCCGT
*Ccl2*	GCAGGTGTCCCAAAGAAGCT	ACAGAAGTGCTTGAGGTGGT
*Tlr4*	TGTATCGGTGGTCAGTGTGC	CAGCTCGTTTCTCACCCAGT
*Vcam1*	GTCAACTGCACGGTCCCTAA	GAGCTTTCCCGGTGTCTTCA

### VSMC function assays

Cell proliferation was assessed using both cell counting and the Cell Counting Kit-8 (CCK-8). Initially, equal numbers of cells were seeded in each well of a cell plate and incubated for 24 h. Subsequently, the cells were treated with 100 μM DHC and 20 ng/mL PDGF-BB for another 24 h. For the cell counting assay, cells were digested with trypsin after 0, 12, 24, and 36 h (four wells at each time point) and counted using an automated cell counter. A cell proliferation line graph was then plotted based on these counts. For the CCK-8 assay, after 24 h of treatment, 10 μL of CCK-8 solution was added to each well, followed by incubation at 37 °C for 2 h in the dark. The absorbance was measured at 450 nm using a microplate reader. The relative cell viability (percentage of control) was calculated using the formula [(As − Ab)/(Ac − Ab)] ×100%, where As is the absorbance of the sample, Ab is the background absorbance, and Ac is the control absorbance.

Cell migration was evaluated using a wound-healing assay. VSMCs were seeded in six-well plates at a density of 1 × 10^6^ cells/well and grown to 100% confluence. A 200 μL pipette tip was then used to create a scratch in the cell monolayer. Then, the wells were rinsed three times with PBS or DMEM to remove detached cells. Cells were treated with 20 ng/mL PDGF-BB and/or 100 μM DHC, and images of the scratch area were captured at 0 h (recorded as A_0_) and after 36 h (recorded as A_36_) of incubation. The percentage of wound closure was calculated using ImageJ software with the formula (A_0_ − A_36_)/A_0_ × 100% to determine the extent of cell migration.

### Hematoxylin and eosin (H&E) staining

The carotid artery samples were embedded with optimal cutting temperature compound. Frozen sections, each with a thickness of 6 μm, were prepared using a cryostat microtome. After fixing with 4% paraformaldehyde (G1101, Servicebio), the sections were stained using an H&E Kit (C0105, Beyotime). Imaging of the stained sections was performed using an upright microscope (Eclipse Ni-U). The ratio of the carotid plaque area to the intimal area was quantitatively analyzed using ImageJ software.

### Immunofluorescence assay

The tissue samples or cells were first fixed using 4% paraformaldehyde. The samples were blocked in a PBS solution containing 0.3% Triton X-100, 10% Western blocking reagent (WBR, 11921673001, Roche), and 5% goat serum for 1 h. Primary antibodies, specifically α-SMA (ab5694 and ab7817, Abcam), F4/80 (30325S, CST), and SM-MHC (21404-1-AP, Proteintech), were applied, and the samples were incubated overnight. The following day, secondary antibodies, including goat anti-mouse Alexa Fluor Plus 488 (A32723, Invitrogen) and goat anti-rabbit Alexa Fluor Plus 555 (A32732, Invitrogen), along with 4′,6-diamidino-2-phenylindole (DAPI, Solarbio) were added, and the samples were incubated at room temperature for 1 h. Imaging was conducted using a Nikon A1R Confocal Microscope. The analysis of fluorescence intensity was performed using ImageJ software.

### Molecular docking and molecular dynamics simulation

The structure of DHC was obtained from PubChem, and the SPTA1 structure was sourced from the Protein Data Bank. Water molecules and ligands were removed from the protein structure using Pymol. Molecular docking was performed using AutoDockTools (version 1.5.7), and molecular dynamics simulations were carried out with GROMACS (version 2022.3). AmberTools 22 was used to apply a force field to the small molecules, with AMBER99SB-ILDN selected as the force field. Water molecules, modeled with the Tip3p water model, served as the solvent. The total charge of the system was neutralized by adding an appropriate amount of Na^+^ ions.

The steepest descent method was applied for energy minimization, followed by equilibration of the system with NVT and NPT ensembles over 100 ps. A subsequent molecular dynamics simulation was conducted, comprising a total of 5,000,000 steps, with each step lasting 2 fs, resulting in a simulation time of 100 ns. Post-simulation analyses included calculations of the root mean square deviation (RMSD), root mean square fluctuation, protein radius of gyration, binding free energy, and free energy landscape of each amino acid trajectory.

### Surface plasmon resonance (SPR)

DHC was diluted to various concentrations with 5% DMSO in a 96-well plate and coupled to the target protein on a CM5 chip, progressing from low to high concentrations. The flow rate was maintained at 30 μL/min, with each measurement lasting 150 s. After each concentration point was tested, the chip was regenerated with a 10 mM glycine hydrochloride solution (pH 2.0) for 5 min. This sequence was repeated until all compound concentrations were analyzed. The data were globally fitted to the 1:1 Langmuir binding model using Biacore Insight software (Cytiva, Marlborough, MA, USA) to determine binding and dissociation constants.

### Data analysis

All data are presented as the mean ± standard error of the mean (mean ± SEM). GraphPad Prism version 8.2.1 was employed for the statistical analysis. Student’s *t*-test was used for comparisons between two groups. One-way ANOVA was conducted for comparisons of three or more groups, and two-way ANOVA was applied to analyze data collected at multiple time points. A value of *P* < 0.05 was considered statistically significant. All experiments were repeated at least three times.

## Results

### Upregulation of contractile phenotype markers in VSMCs treated with DHC according to RNA-seq analysis

We first analyzed the effects of different DHC concentrations on VSMCs using a CCK-8 assay. A DHC concentration below 100 μM exhibited no cytotoxicity on VSMCs, but when the DHC concentration reached 200 μM, it exhibited significant cytotoxicity (Fig. S1). The above results provide valuable reference information for the concentration selection of DHC in this study. In addition, based on the previous results of our research group [19], we selected 100 μM DHC to treat VSMCs.

**Fig. 1:**
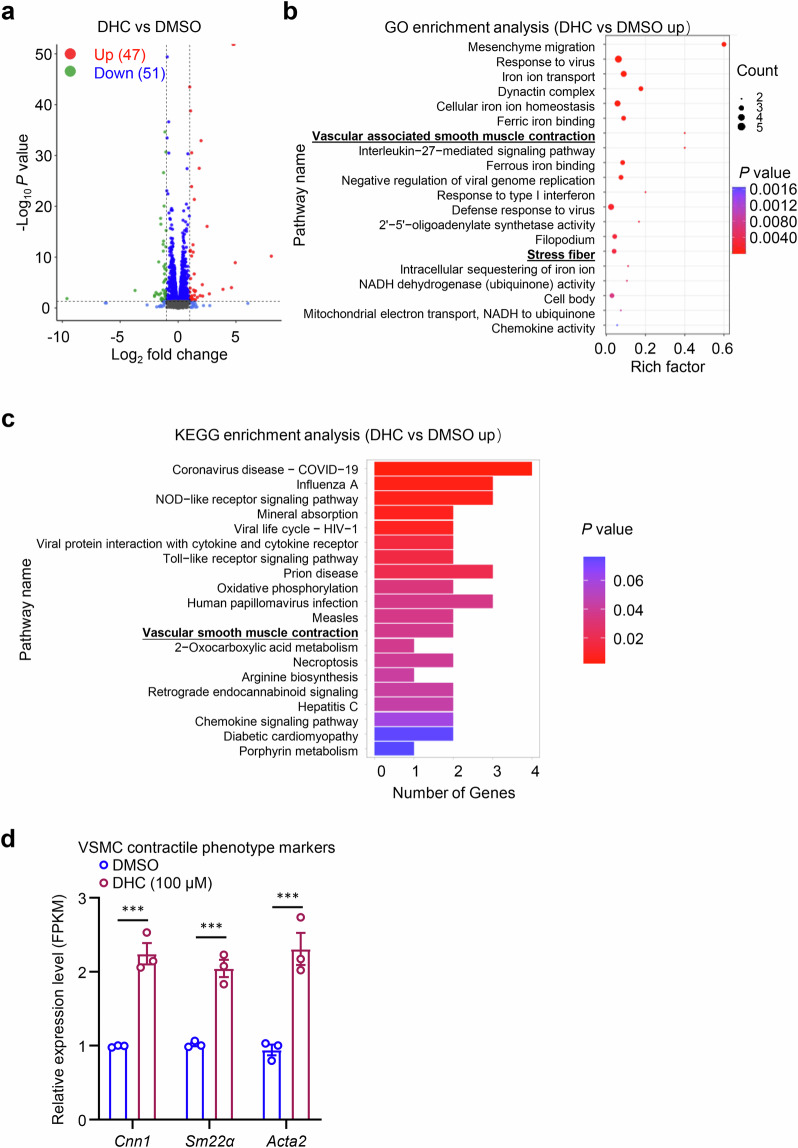
RNA-sequencing (RNA-seq) analysis shows that dehydrocorydaline (DHC) enhances the expression of contractile phenotype-related genes in rat vascular smooth muscle cells (VSMCs). Rat VSMCs were exposed to either dimethyl sulfoxide (DMSO) or 100 μM DHC for 24 h, followed by RNA-seq analysis (*n* = 3). **a** A volcano plot shows differentially expressed genes, with red and green dots representing significantly upregulated (47) and downregulated genes (51), respectively (*P* < 0.05, |log2 fold change | > 1). **b** The top 20 enriched upregulated Gene Ontology pathways. The dot size represents the number of differentially expressed genes in the pathway, and the dot colors correspond to various *P* values. **c** The top 20 enriched upregulated Kyoto Encyclopedia of Genes and Genomes pathways. The dot size represents the number of differentially expressed genes in the pathway, and the dot colors correspond to different ranges of *P* values. **d** Fragments per kilobase of transcript per million mapped reads (FPKM) values of markers related to VSMC phenotypic switching among differentially expressed genes, including Calponin 1 (*Cnn1*), smooth muscle 22α (*Sm22α*), and alpha-smooth muscle actin (*Acta2*). Data are presented as the mean ± SEM. ***P < 0.001, analyzed using unpaired *t*-tests.

To investigate the impact of DHC on VSMCs, rat VSMCs were treated with 100 μM DHC for 24 h, and total RNA was extracted for RNA-seq transcriptome analysis. Following DHC treatment, 47 genes were significantly upregulated, while 51 were downregulated (Fig. 1a). Gene Ontology (GO) analysis illustrated that the top 20 upregulated genes were associated with smooth muscle contraction and stress fiber pathways (Fig. 1b). Kyoto Encyclopedia of Genes and Genomes analysis corroborated the enrichment of the vascular smooth muscle contraction pathway (Fig. 1c). Subsequent analysis of upregulated genes revealed a significant increase in the expression of VSMC contractile phenotype markers, including *Cnn1*, *Sm22α*, and *Acta2* (Fig. 1d), suggesting a potential role of DHC in VSMC phenotypic switching.

### DHC maintains the VSMC contractile phenotype

**Fig. 2:**
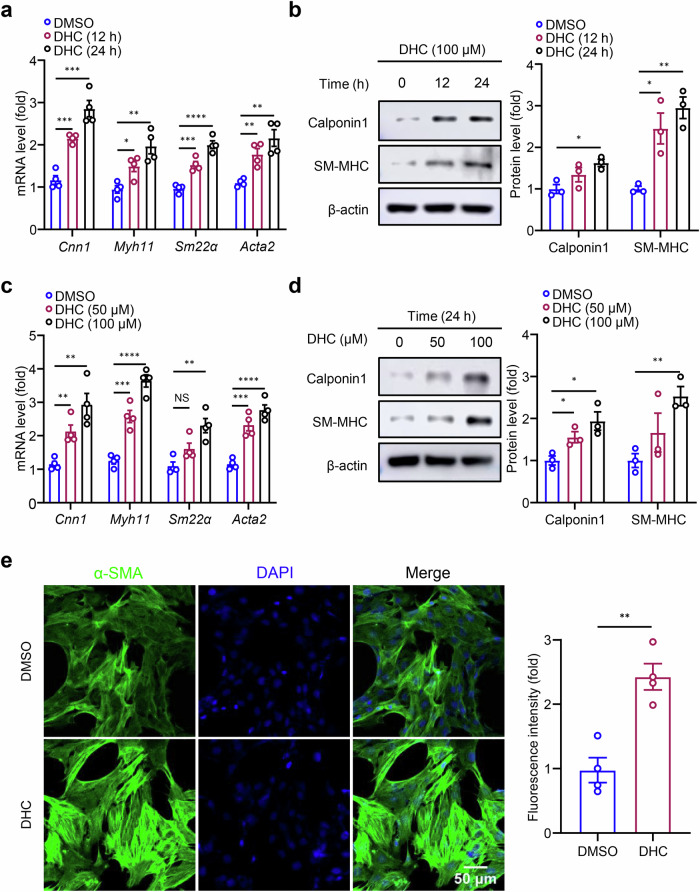
DHC maintains the VSMC contractile phenotype. **a**, **b** Rat VSMCs were treated with 100 μM DHC for 0, 12, and 24 h. **a** mRNA levels of contractile markers (*Cnn1*, smooth muscle myosin heavy chain (*Myh11*), *Sm22α*, and *Acta2*) were detected by quantitative reverse transcription polymerase chain reaction (RT-qPCR) (*n* = 4). **b** Protein levels of contractile markers (Cnn1 and smooth muscle myosin heavy chain, SM-MHC) were detected by Western blotting (*n* = 3). **c**, **d** Rat VSMCs were treated with 0, 50, and 100 μM DHC for 24 h. **c** mRNA levels of contractile markers (*Cnn1*, *Myh11*, *Sm22α*, and *Acta2*) were assessed by RT-qPCR (*n* = 4). **d** Protein levels of contractile markers (Cnn1 and SM-MHC) were determined by Western blotting (*n* = 3). **e** α-SMA (green) expression in rat VSMCs treated with DMSO or 100 μM DHC for 24 h was detected by cyto-immunofluorescence, and 4′,6-diamidino-2-phenylindole (DAPI) (blue) indicates the nucleus (*n* = 4, scale = 50 μm). Data are presented as the mean ± SEM. *P < 0.05, **P < 0.01, ***P < 0.001, and ****P < 0.0001, analyzed using unpaired *t*-tests.

To elucidate the effect of DHC on VSMC phenotypic switching, rat VSMCs were treated with either DMSO or 100 μM DHC for 12 or 24 h. Expression levels of VSMC phenotypic switching-related markers were detected by RT-qPCR. The results demonstrated a time-dependent upregulation of mRNA levels of VSMC contractile phenotype markers (*Cnn1*, *Myh11*, *Sm22α*, and *Acta2*) with increasing DHC treatment time (Fig. 2a). Parallel Western blotting analyses revealed a gradual upregulation of VSMC contractile phenotype markers (Cnn1 and SM-MHC) with prolonged DHC treatment (Fig. 2b). Consistent results were obtained when VSMCs were treated with 0, 50, and 100 μM DHC for 24 h (Fig. 2c, d), confirming that DHC promotes the expression of contractile markers in rat VSMCs in a time- and concentration-dependent manner. Cyto-immunofluorescence of the intracellular contractile marker α-SMA supported these findings, demonstrating increased protein expression 24 h after DHC treatment (Fig. 2e). The above results confirm that DHC plays a role in maintaining the rat VSMC contractile phenotype.

In addition, VSMC phenotypic switching is often associated with a change in inflammatory cytokines [23]. Thus, we also investigated the impact of DHC on inflammatory factors. Rat VSMCs were treated with DHC as indicated. qPCR was used to determine the gene expression levels of the inflammatory factors *Cd68*, CC chemokine ligand 2 (*Ccl2*), Toll-like receptor 4 (*Tlr4*), and vascular cell adhesion molecule 1 (*Vcam1*). DHC reduced the mRNA levels of *Cd68*, *Ccl2*, *Tlr4*, and *Vcam1* (Fig. S2a, c). Among these, VCAM1, the factor most significantly affected by DHC, was selected for further validation at the protein level. The results demonstrated that DHC effectively inhibited VCAM1 protein expression (Fig. S2b, d). Taken together, these findings suggest that DHC reduces the expression of inflammatory factors.

### DHC inhibits the PDGF-BB-induced VSMC secretory phenotype transition in vitro

**Fig. 3:**
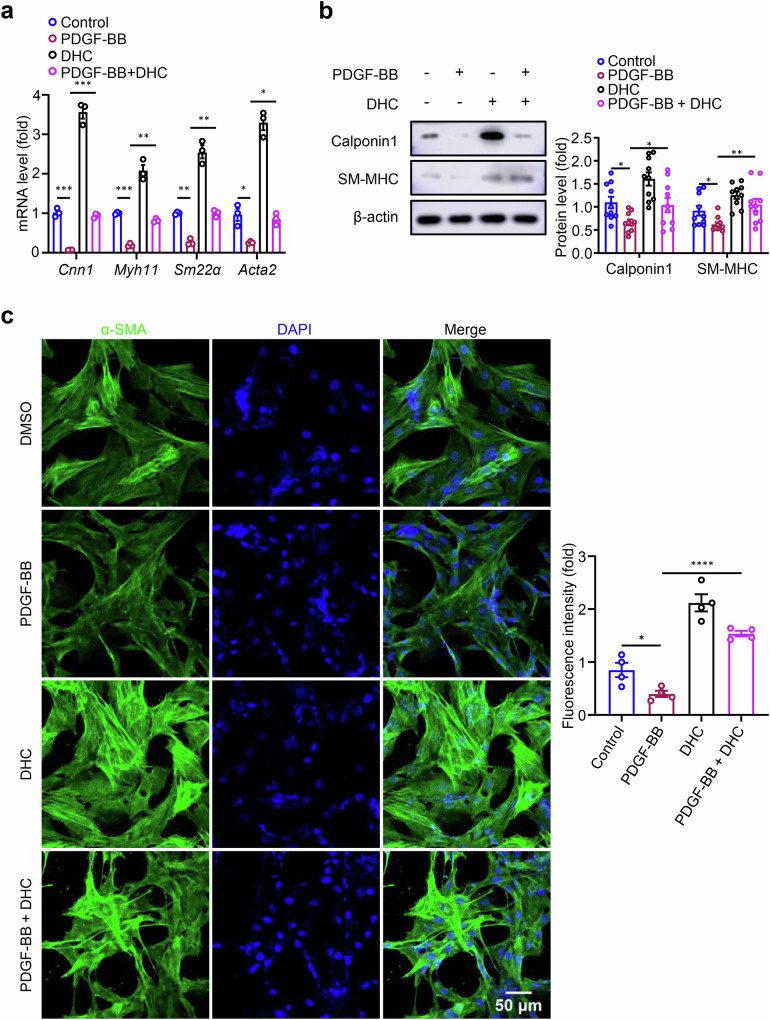
DHC suppresses the platelet-derived growth factor-BB (PDGF-BB)-induced VSMC secretory phenotype transition in vitro. **a**–**c** Rat VSMCs were exposed to 100 μM DHC and/or 20 ng/mL PDGF-BB for 24 h. **a** mRNA levels of contractile markers (*Cnn1*, *Myh11*, *Sm22α*, and *Acta2*) were assessed by RT-qPCR (*n* = 3). **b** Protein levels of contractile markers (Cnn1 and SM-MHC) were detected by Western blotting (*n* = 10). **c** Protein levels of α-SMA (green) were detected by immunofluorescence, and DAPI (blue) indicates the nucleus (*n* = 4, scale = 50 μm). Data are presented as the mean ± SEM. *P < 0.05, **P < 0.01, ***P < 0.001, and ****P < 0.0001, analyzed using one-way ANOVA.

PDGF-BB is recognized as an important factor in promoting VSMC phenotypic switching and is widely used to construct dedifferentiated VSMC phenotypes for in vitro experiments. Inhibition of PDGF-BB signaling by blocking PDGF receptors can reduce fibrous cap formation in *Apoe*^*−/−*^ mice fed a high-fat diet [24]. Therefore, we further observed the effect of DHC on the phenotypic switching of VSMCs stimulated by PDGF-BB. Cells were pre-treated with 100 μM DHC for 15 min and then co-treated with 20 ng/mL PDGF-BB for 24 h. The effect of DHC on VSMC phenotype marker expression was detected by RT-qPCR and Western blotting. Compared with those in the control group, the mRNA and protein expression levels of contractile phenotype markers were significantly downregulated after PDGF-BB treatment. This finding indicates that PDGF-BB successfully induced the transition of VSMCs to a secretory phenotype. However, DHC reversed the downregulation of contractile phenotypic markers induced by PDGF-BB (Fig. 3a, b). The cyto-immunofluorescence results showed that the fluorescence intensity of α-SMA expression in the DHC combined with PDGF-BB treatment group was significantly higher than that in the PDGF-BB alone treatment group (Fig. 3c). Together, these results indicate that DHC can reverse PDGF-BB-induced VSMC secretory phenotypic switching and maintain the contractile phenotype of cells.

### DHC inhibits PDGF-BB-induced VSMC proliferation and migration

**Fig. 4:**
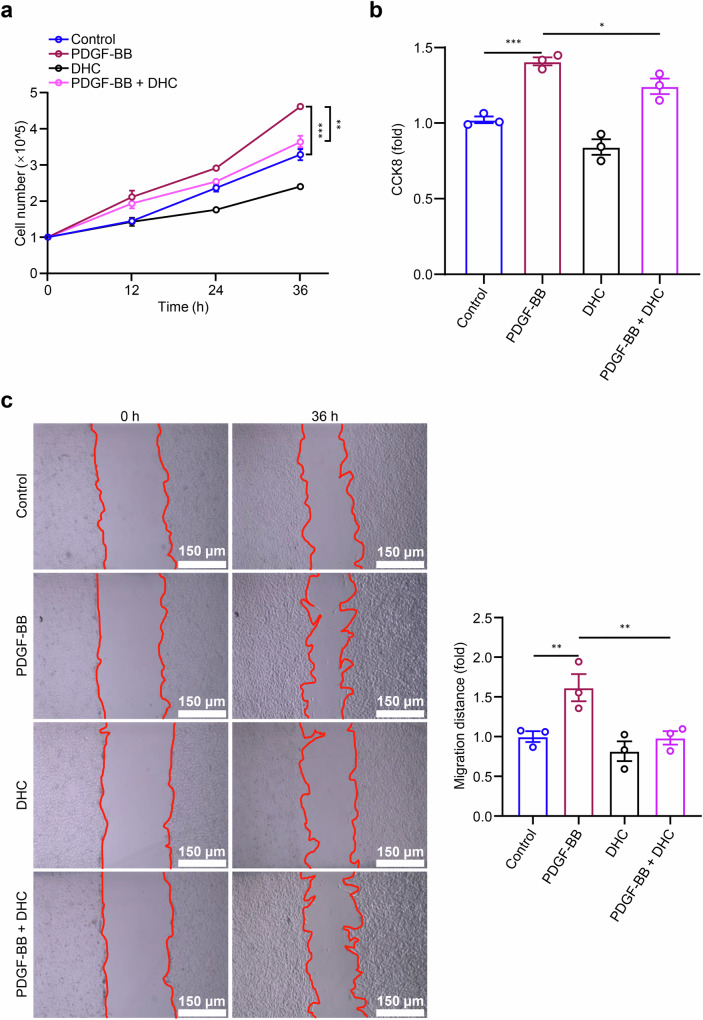
DHC inhibits PDGF-BB-induced VSMC proliferation and migration. **a**–**c** VSMCs were treated with 100 μM DHC and/or 20 ng/mL PDGF-BB for 36 h. The effects of PDGF-BB and DHC on VSMC proliferation were determined by cell counting **a** and a Cell Counting Kit-8 (CCK-8) assay (**b**), *n* = 3. **c** The effects of PDGF-BB and DHC on VSMC migration were examined using a wound healing assay (*n* = 3, scale = 150 μm). Data are presented as the mean ± SEM. *P < 0.05, **P < 0.01, and ***P < 0.001, analyzed using two-way ANOVA (**a**) and one-way ANOVA (**b**, **c**).

The transition of VSMCs from a contractile phenotype to a secretory phenotype is marked by enhanced proliferation and migration. We next sought to determine whether DHC could effectively inhibit VSMC proliferation and migration in vitro. Utilizing cell counting and the CCK-8 kit, we assessed cell proliferation and observed a significant increase following the induction of VSMC phenotype switching by PDGF-BB (Fig. 4a, b). Remarkably, the introduction of 100 μM DHC reversed this proliferation (Fig. 4a, b). Additionally, a wound healing assay revealed that DHC inhibited PDGF-BB-induced VSMC migration, as evidenced by a reduction in cell migration compared with that in the PDGF-BB alone treatment group after 36 h (Fig. 4c). These findings confirm that DHC effectively inhibits PDGF-BB-induced VSMC proliferation and migration.

### DHC attenuates carotid atherosclerosis in Apoe^−/−^ mice

**Fig. 5:**
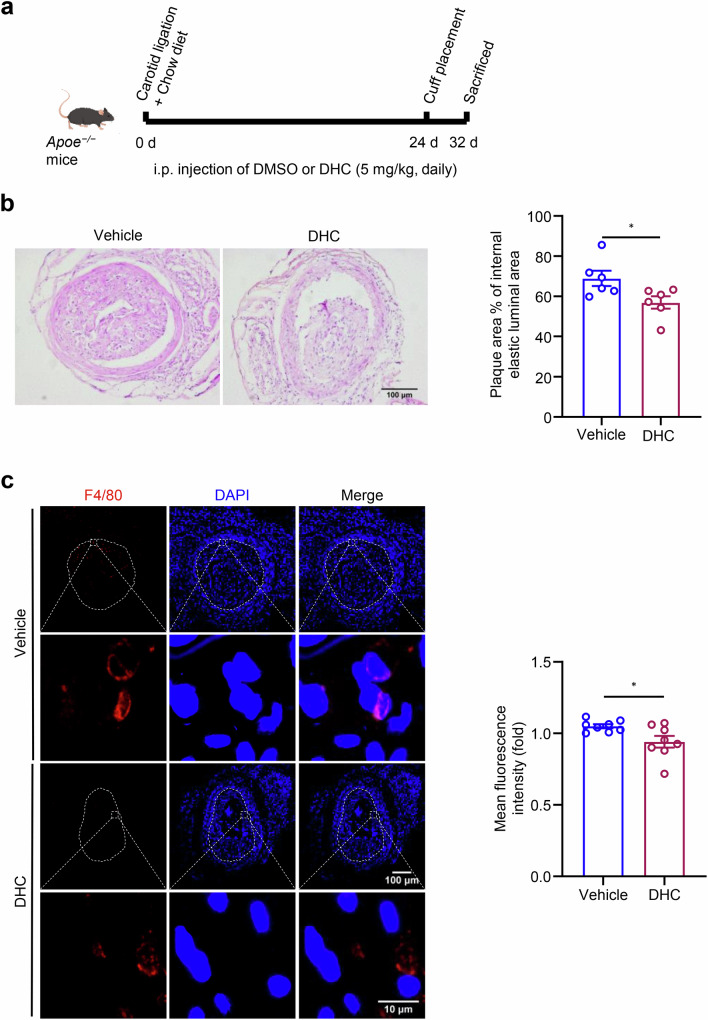
DHC attenuates atherosclerosis in *Apoe*^*−/−*^ mice. **a** The strategy for an *Apoe*^*−/−*^ mouse model with carotid artery injury. **b** Carotid atherosclerotic plaque lesions evaluated by hematoxylin and eosin staining (*n* = 6/group, scale = 100 μm). **c** Detection of macrophage infiltration in carotid plaques by F4/80 (red) immunofluorescence staining, with DAPI (blue) indicating the nucleus (*n* = 8/group, scale = 100 μm/10 μm). Data are presented as the mean ± SEM. *P < 0.05, analyzed using unpaired *t*-tests.

To demonstrate the role of DHC in atherosclerotic plaques, *Apoe*^*−/−*^ mice were randomly divided into two groups. The RCCA was ligated for 28 days and a cuff was placed for 4 days to construct a carotid artery injury model. One group received intraperitoneal injections of DMSO (vehicle group), while the other group received 5 mg/kg DHC (DHC treatment group) (Fig. 5a). H&E staining of frozen sections revealed varying degrees of plaques in the right carotid arteries of both groups following carotid artery injury. However, DHC treatment attenuated carotid atherosclerosis (Fig. 5b). Immunofluorescence staining of the macrophage marker F4/80 indicated reduced macrophage infiltration in the DHC treatment group compared with that in the vehicle group (Fig. 5c). In summary, our results suggest that DHC mitigates carotid atherosclerosis in *Apoe*^*−/−*^ mice.

### DHC maintains the VSMC contractile phenotype of carotid plaques in Apoe^−/−^ mice

**Fig. 6:**
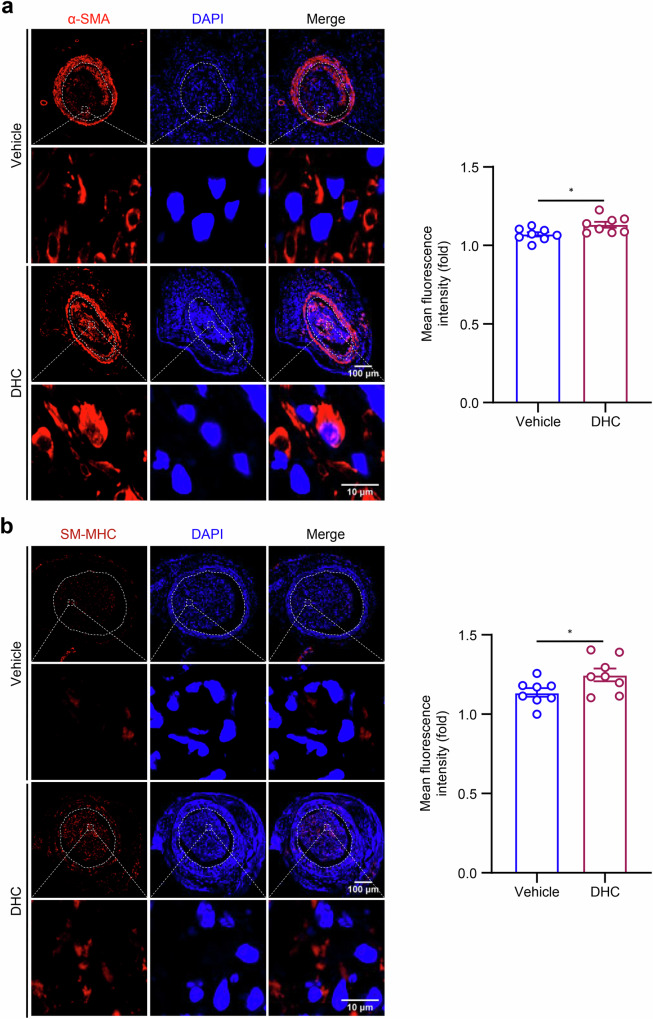
DHC maintains the VSMC contractile phenotype in the carotid plaques of *Apoe*^*−/−*^ mice. **a** Immunofluorescence detection of α-SMA (red) in carotid plaques, with DAPI (blue) indicating the nucleus (*n* = 8/group, scale = 50 μm/10 μm). **b** Immunofluorescence detection of SM-MHC (red) in carotid plaques, with DAPI (blue) indicating the nucleus (*n* = 8/group, scale = 100 μm or 10 μm). Data are presented as the mean ± SEM. *P < 0.05, analyzed using unpaired *t*-tests.

The transition of VSMCs from a contractile to a secretory phenotype is a key factor in the formation of atherosclerotic plaques. While the role of DHC in plaques has been previously demonstrated, its effect on regulating VSMC phenotypic switching in carotid plaques remains unknown. To explore this effect, we performed immunofluorescence staining and statistical analysis of the contractile phenotype markers α-SMA and SM-MHC in carotid artery sections. Our findings indicated that, compared with the vehicle group, the average fluorescence intensity of these contractile phenotype markers was increased in carotid plaques of *Apoe*^*−/−*^ mice treated with DHC (Fig. 6a, b). This result suggests that DHC can maintain the VSMC contractile phenotype in *Apoe*^*−/−*^ mice.

### DHC sustains the contractile phenotype of rat VSMCs through upregulation of Spta1 expression

**Fig. 7:**
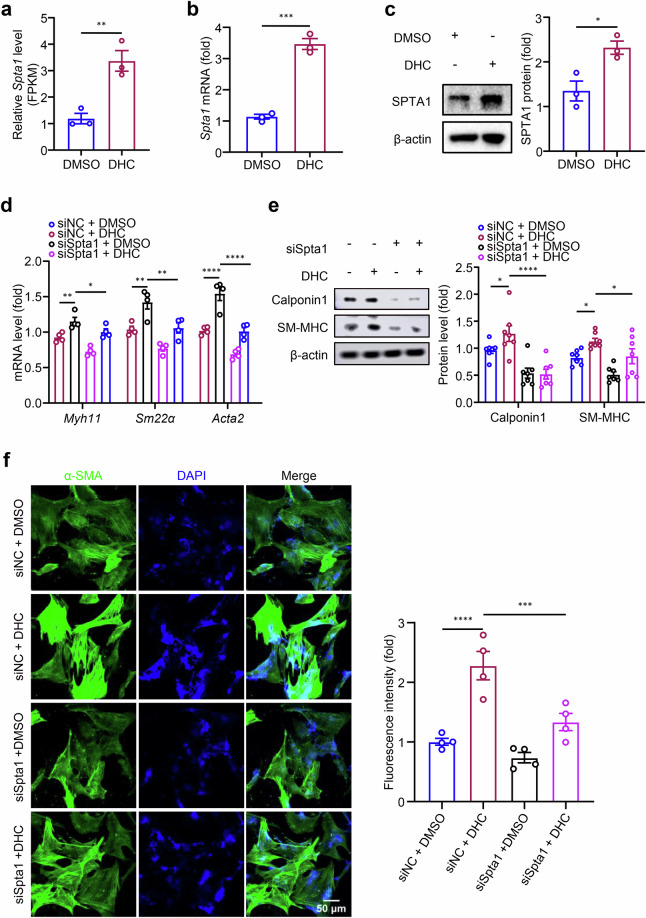
DHC sustains the contractile phenotype of rat VSMCs by upregulating spectrin alpha, erythrocytic 1 (Spta1) expression. **a**–**c** Rat VSMCs were treated with DMSO or 100 μM DHC for 24 h. **a** FPKM values of Spta1 from RNA-seq analysis (*n* = 3). **b** Detection of Spta1 mRNA level using RT-qPCR (*n* = 3). **c** Detection of SPTA1 protein level by Western blotting (*n* = 3). **d**, **e** Rat VSMCs were transfected with siNC or siSpta1 for 24 h and then treated with DMSO or 100 μM DHC for 24 h. **d** mRNA levels of contractile markers (*Myh11*, *Sm22α*, and *Acta2*) were assessed using RT-qPCR (*n* = 4). **e** Protein levels of contractile markers (Cnn1 and SM-MHC) were detected by Western blotting (*n* = 7). **f** Expression of α-SMA (green) in rat VSMCs was determined by immunofluorescence, with DAPI (blue) indicating the nucleus (*n* = 4, scale = 50 μm). Data are presented as the mean ± SEM. *P < 0.05, **P < 0.01, ***P < 0.001, and ****P < 0.0001, analyzed using unpaired *t*-tests (**a** to **c**) and one-way ANOVA (**d** to **f**).

To further investigate the specific mechanism by which DHC maintains the VSMC contractile phenotype, the top 10 upregulated and top 10 downregulated differentially expressed genes were queried in this study, and it was found that the differentially expressed genes *Spectrin*, *alpha*, and *erythrocytic 1* (*Spta1*) located at the Position 8 of the upregulated DEGs were significantly induced by DHC (Fig. 7a). Spta1 is a member of the molecular scaffold protein (spectrin and hemopoietin) family and is a cytoskeletal protein on the inner side of the cell membrane. The gene annotation results were related to “actin binding” and “actin cytoskeleton organization”, including GO: 0003779 (molecular_function, actin binding), GO: 0005737 (cellular_component, cytoplasm), GO: 0030036 (biological_process, actin cytoskeleton organization), GO: 0030863 (cellular_component, cortical cytoskeleton), and GO: 0030864 (cellular_component, cortical actin cytoskeleton). In recent years, studies have uncovered the role of Spta1 in smooth muscle cell phenotypic switching. Chen et al. reported that transfection of siSpta1 into corpus cavernosum smooth muscle cells (CCSMCs) led to a marked decrease in the expression of α-SMA, a contractile protein in CCSMCs, suggesting Spta1 maintains the smooth muscle cell contractile phenotype [25].

To explore whether Spta1 mediates the effects of DHC on the phenotypic switching of VSMCs, we first examined the expression level of *Spta1* mRNA after DHC treatment. DHC significantly induced *Spta1* mRNA (Fig. 7b). Moreover, Western blotting showed that DHC upregulated the protein level of SPTA1 compared with that in the control group (Fig. 7c). Next, we treated siSpta1-transfected VSMCs with 100 μM DHC. Compared with the siNC group, the Spta1 protein level in the siSpta1 knockdown group was significantly downregulated, indicating the reliability of siSpta1 knockdown efficiency (Fig. S3). Specifically, compared with the DHC group, the DHC combined with siSpta1 treatment group showed a reduction in mRNA levels of *Myh11*, *Sm22α*, and *Acta2* and protein levels of Cnn1, SM-MHC, and α-SMA, indicating that Spta1 mediates the effects of DHC (Fig. 7d–f). Taken together, these results suggested that DHC may promote the switching of rat VSMCs to a contractile phenotype by upregulating Spta1 expression.

We finally analyzed the interaction between SPTA1 and DHC using molecular docking, molecular dynamics simulation, and SPR analyses. The molecular docking results indicated hydrogen bonds were formed between DHC and SPTA1 through the ARG118 amino acid residue, with a calculated binding energy of −6.3 kcal/mol (Fig. S4a). Typically, binding energy less than −5 kcal/mol suggests a binding affinity between a small molecule and a protein, supporting a measurable interaction between DHC and SPTA1. The RMSD is a commonly used metric to assess molecular structural differences, and it illustrates fluctuations in protein conformation. As shown in Fig. S4b, noticeable fluctuations of the RMSD occurred in the early stages; however, after 19 ns, the fluctuation range of the SPTA1-DHC complex remained below 0.2 nm, indicating that the conformation of SPTA1 remained stable after binding with DHC. The root mean square fluctuation curve reflected amino acid residue fluctuations, with the SPTA1-DHC complex showing high flexibility between residues 0 and 45 and between residues 77 and 95 (Fig. S4c). The radius of gyration, which reflects binding tightness and protein folding, stabilized after 20 ns, suggesting a compact, stable complex conformation (Fig. S4d). The Gibbs energy landscape illustrated the stability of the complex, with PC1 (RMSD) and PC2 (radius of gyration) indicating steady-state structures. The region of minimal free energy, shown in blue, highlighted the conformational stability of the complex (Fig. S4e). Hydrogen bonds and hydrophobic interactions are crucial to maintain protein conformation; analysis indicated zero to three hydrogen bonds over a 0–100 ns period, supporting the stability of the SPTA1-DHC complex (Fig. S4f). Energy decomposition analysis revealed the role of key residues, with ARG118 and HIS132 being crucial for stabilizing the binding interaction between SPTA1 and DHC (Fig. S4g). The binding free energy of the complex, including contributions from van der Waals, electrostatic, polar solvation, and non-polar solvation energies, was calculated as −21.31 kcal/mol, indicating robust binding. Finally, the SPR experiment showed that DHC binds to SPTA1 with a *K*_d_ of 2.18 µmol/L (Fig. S4h). Collectively, these results suggest an interaction between SPTA1 and DHC.

## Discussion

**Fig. 8:**
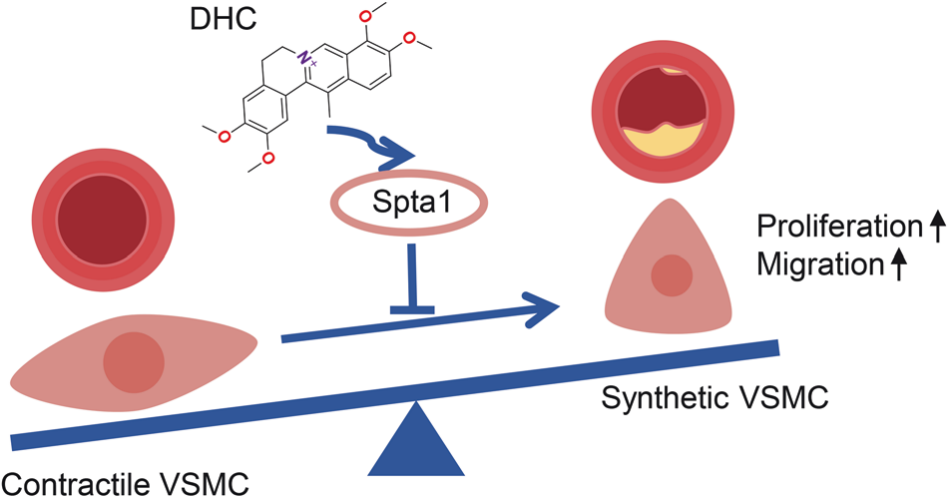
Schematic illustration of the effects of DHC on VSMCs and atherosclerosis. DHC from *Rhizoma Corydalis* promotes the contractile phenotype of VSMCs by upregulating Spta1, thereby inhibiting VSMC proliferation and migration and reducing atherosclerotic plaques in *Apoe*^−/−^ mice, suggesting its potential in treating atherosclerotic cardiovascular disease.

In our current study, we successfully demonstrated that DHC, an alkaloid extracted from *Rhizoma Corydalis*, maintains the contractile phenotype of VSMCs and inhibits PDGF-BB-induced VSMC phenotypic switching. Furthermore, treatment of *Apoe*^*−/*−^ mice with DHC resulted in reduced carotid plaque areas and an increased presence of contractile VSMCs. We also discovered that Spta1 plays an essential role in mediating the suppressive effect of DHC on VSMC phenotypic switching (Fig. 8).

Our results demonstrated that DHC can maintain the contractile phenotype of VSMCs, as evidenced by the upregulation of contractile phenotype markers. This process is crucial because VSMCs in a contractile state play a role in regulating vascular diameter and blood flow distribution. When VSMCs are damaged, contractile VSMCs can temporarily modify their phenotype to adopt a highly synthetic state. However, the consequences can be illustrated from two aspects. First, after damage and external stimulation subside, the local vascular environment returns to normal, and the VSMCs regain their contractile phenotype characteristics [26]. Second, lineage tracing and single-cell sequencing studies have elucidated that contractile VSMCs possess the ability to transition into diverse dedifferentiated phenotypes, including osteogenic, macrophage-like, foam cell, myofibroblast-like, osteochondral-like, and mesenchymal stem-like phenotypes [27]. Notably, a shared feature among these phenotypes is the downregulation of contractile phenotype markers, facilitating cellular adaptation to external stimuli and damage. Our RNA-seq analysis showed, for the first time, that DHC could significantly upregulate contractile phenotype marker expression in VSMCs and further demonstrated the important role of DHC in maintaining the VSMC contractile phenotype across various time and concentration gradients in vitro. Our investigation revealed, for the first time, that DHC induces upregulation of VSMC contractile phenotype markers in a time- and concentration-dependent manner. More importantly, DHC maintained the contractile phenotype of VSMCs in *Apoe*^*−/−*^ mice.

Many alkaloids have been shown to regulate VSMC proliferation and migration or phenotypic switching. For example, sinomenine has been shown to attenuate PDGF-BB-induced proliferation, migration, and dedifferentiation of rat VSMCs cultured in vitro and neointimal formation in the carotid artery in wire-injured mice [28]. Currier et al. reported that the severity of restenosis at 4 weeks after balloon angioplasty of the iliac artery in animals treated with high-dose colchicine was moderately reduced [29], suggesting a potential role in preventing restenosis following percutaneous coronary revascularization. Li et al. found that alkaloids from Nelumbinis Plumula (AFNP) could significantly increase α-SMA expression, rescue angiotensin-II-induced VSMC phenotypic switching, inhibit proliferation and migration, and significantly ameliorate aortic remodeling [30]. Seahyoung Lee noted that berberine inhibited angiotensin-II and heparin-binding epidermal growth factor-induced rat VSMC proliferation and migration in vitro by delaying or partially inhibiting Akt activation and improved neointimal formation in vivo after balloon injury [31]. Wang et al. noted that rutaecarpine could prevent VSMC dysfunction by inhibiting ox-LDL-induced VSMC proliferation, migration, and phenotypic switching [32]. We found that DHC, an alkaloid, could inhibit both the proliferation and migration of VSMCs. VSMC phenotypic switching is closely related to plaque progression. We constructed a model of carotid artery injury and administered daily intraperitoneal injections of DHC. The carotid artery section staining results revealed that DHC reduced plaque area and macrophage infiltration in *Apoe*^*−/−*^ mice, consistent with our previously published findings [19].

VSMC proliferation and migration promote the pathogenesis and development of atherosclerosis. VSMC migration requires the degradation of various components of the matrix, including the basilar membrane and the elastic layer, which may occur through the matrix metalloproteinase (MMP) family. In particular, the gelatinases MMP-2 and MMP-9, with similar substrate affinities in vitro, are recognized for their role in degrading short collagen, interstitial collagen, and elastin [33]. This degradation contributes to VSMC migration, proliferation, and the acceleration of intimal thickening and vascular wall remodeling in atherosclerosis. We found that DHC significantly attenuated PDGF-BB-induced VSMC proliferation and migration. This anti-proliferative and anti-migratory effect aligns with the anti-atherosclerotic role of DHC.

This study identified Spta1 as a key mediator of DHC in maintaining the contractile phenotype of VSMCs. Spta1 knockdown inhibited the regulatory effect of DHC on phenotypic switching, implicating Spta1 as a potential target for the treatment of CVDs related to phenotypic switching. Furthermore, a noteworthy revelation from this study was that Spta1 is a key mediator through which DHC maintains the contractile phenotype of rat VSMCs. Spectrin, initially characterized from human erythrocyte ghosts in 1968 [34], is a filamentous protein that predominantly exists as heterotetramers composed of various α and β subunit isomers, forming a polygonal network structure beneath the erythrocyte membrane. Spta1 is a protein-coding gene that encodes human erythroid α-hematopoietin. Mutations in this gene can lead to severe anemia, which is characterized by an abnormal erythrocyte shape and increased membrane fragility. A subsequent study showed that compared with healthy individuals, hypertensive patients exhibited decreased levels of α-spectrin (Spta1) and β-spectrin (Sptb) and increased glucose transporter (GLUT1) levels [35]. Spectrin has been reported to influence the growth and development of cells and tissues by regulating the Hippo-YAP signaling pathway [36, 37], and it inhibits the phenotypic switching and proliferation of CCSMCs, resulting in erectile dysfunction in rats. Our study demonstrated that DHC significantly upregulated the mRNA and protein levels of Spta1, which regulates the inhibitory effect of DHC on VSMC phenotypic switching, suggesting Spta1 as a novel target for CVDs caused by phenotypic switching.

Using a model of carotid artery injury in *Apoe*^*−/−*^ mice, we found that DHC treatment resulted in a reduced plaque area, diminished macrophage infiltration, and an increased presence of contractile VSMCs. This finding suggested that DHC has a protective effect against carotid atherosclerosis. Although our prior work demonstrated the role of DHC in mitigating atherosclerotic lesions and regulating aortic compliance, its specific impact on plaque stability remained unexplored. Therefore, we employed a carotid artery injury model, following established methods, to evaluate the effect of DHC on plaques based on the plaque lesion area, macrophage infiltration, and phenotypic switching. The carotid plaques of *Apoe*^*−/−*^ mice in the DHC treatment group presented fewer lesions, less macrophage infiltration, and more contractile VSMCs than those of control mice. These findings indicate that DHC can attenuate carotid atherosclerosis in *Apoe*^*−/−*^ mice and maintain the VSMC contractile phenotype in vivo.

The study concludes that DHC has a significant role in maintaining the VSMC contractile phenotype, inhibiting phenotypic switching, and attenuating atherosclerosis in *Apoe*^*−*/−^ mice. The identification of Spta1 as a key mediator adds to the understanding of the molecular mechanisms involved. The findings not only contribute to the understanding of the potential therapeutic effects of DHC but also open avenues for the clinical application of Chinese herbal medicine containing DHC in CVDs. However, we acknowledge that all data presented in this study were derived from rat VSMCs, which may limit the direct applicability of our findings to human aortic VSMCs. Future studies are required to validate the role of DHC in human aortic VSMCs, which will be crucial to confirm its therapeutic potential in human atherosclerosis and its broader clinical relevance.

## Supplementary information

Supplementary Figure S1

Supplementary Figure S2

Supplementary Figure S3

Supplementary Figure S4

Uncropped blots

Supplementary figure legend

## References

[1] Joseph P, Leong D, Mckee M, Anand SS, Schwalm JD, Teo K, et al. Reducing the global burden of cardiovascular disease, Part 1: the epidemiology and risk factors. Circ Res. 2017;121:677–94. 28860318 10.1161/CIRCRESAHA.117.308903

[2] Baigent C, Blackwell L, Emberson J, Holland LE, Reith C, Bhala N, et al. Efficacy and safety of more intensive lowering of LDL cholesterol: a meta-analysis of data from 170,000 participants in 26 randomised trials. Lancet. 2010;376:1670–81. 21067804 10.1016/S0140-6736(10)61350-5 PMC2988224

[3] Ridker PM, Everett BM, Thuren T, Macfadyen JG, Chang WH, Ballantyne C, et al. Antiinflammatory therapy with Canakinumab for atherosclerotic disease. N Engl J Med. 2017;377:1119–31. 28845751 10.1056/NEJMoa1707914

[4] Tardif JC, Kouz S, Waters DD, Bertrand OF, Diaz R, Maggioni AP, et al. Efficacy and safety of low-dose colchicine after myocardial infarction. N Engl J Med. 2019;381:2497–505. 31733140 10.1056/NEJMoa1912388

[5] Yin C, Ge Z, Yuan J, Chen Y, Tang Y, Xiang Y, et al. NEAT1 regulates VSMC differentiation and calcification in as long noncoding RNA NEAT1 enhances phenotypic and osteogenic switching of vascular smooth muscle cells in atherosclerosis via scaffolding EZH2. Am J Physiol Cell Physiol. 2024;326:C1721–c1734. 38646788 10.1152/ajpcell.00587.2023 PMC11371316

[6] Furmanik M, Chatrou M, Van Gorp R, Akbulut A, Willems B, Schmidt H, et al. Reactive oxygen-forming Nox5 links vascular smooth muscle cell phenotypic switching and extracellular vesicle-mediated vascular calcification. Circ Res. 2020;127:911–27. 32564697 10.1161/CIRCRESAHA.119.316159

[7] Mao N, Gu T, Shi E, Zhang G, Yu L, Wang C. Phenotypic switching of vascular smooth muscle cells in animal model of rat thoracic aortic aneurysm. Interact Cardiovasc Thorac Surg. 2015;21:62–70. 25829166 10.1093/icvts/ivv074

[8] Wang ZY, Cheng J, Liu B, Xie F, Li CL, Qiao W, et al. Protein deglycase DJ-1 deficiency induces phenotypic switching in vascular smooth muscle cells and exacerbates atherosclerotic plaque instability. J Cell Mol Med. 2021;25:2816–27. 33501750 10.1111/jcmm.16311 PMC7957272

[9] Talasila A, Yu H, Ackers-Johnson M, Bot M, Van Berkel T, Bennett MR, et al. Myocardin regulates vascular response to injury through miR-24/-29a and platelet-derived growth factor receptor-β. Arterioscler Thromb Vasc Biol. 2013;33:2355–65. 23825366 10.1161/ATVBAHA.112.301000

[10] Ding Y, Zhang M, Zhang W, Lu Q, Cai Z, Song P, et al. AMP-activated protein kinase alpha 2 deletion induces VSMC phenotypic switching and reduces features of atherosclerotic plaque stability. Circ Res. 2016;119:718–30. 27439892 10.1161/CIRCRESAHA.116.308689 PMC6265658

[11] Kubo M, Matsuda H, Tokuoka K, Ma S, Shiomoto H. Anti-inflammatory activities of methanolic extract and alkaloidal components from Corydalis tuber. Biol Pharm Bull. 1994;17:262–5. 7515744 10.1248/bpb.17.262

[12] Wang C, Wang S, Fan G, Zou H. Screening of antinociceptive components in Corydalis yanhusuo W.T. Wang by comprehensive two-dimensional liquid chromatography/tandem mass spectrometry. Anal Bioanal Chem. 2010;396:1731–40. 20101504 10.1007/s00216-009-3409-1

[13] Yin ZY, Li L, Chu SS, Sun Q, Ma ZL, Gu XP. Antinociceptive effects of dehydrocorydaline in mouse models of inflammatory pain involve the opioid receptor and inflammatory cytokines. Sci Rep. 2016;6:27129. 27272194 10.1038/srep27129 PMC4895225

[14] Matsuda H, Tokuoka K, Wu J, Shiomoto H, Kubo M. Inhibitory effects of dehydrocorydaline isolated from Corydalis Tuber against type I-IV allergic models. Biol Pharm Bull. 1997;20:431–4. 9145224 10.1248/bpb.20.431

[15] Hu H, Dong Z, Wang X, Bai L, Lei Q, Yang J, et al. Dehydrocorydaline inhibits cell proliferation, migration and invasion via suppressing MEK1/2-ERK1/2 cascade in melanoma. Onco Targets Ther. 2019;12:5163–75. 31456643 10.2147/OTT.S183558 PMC6620435

[16] Huang Y, Huang H, Wang S, Chen F, Zheng G. Dehydrocorydaline inhibits the tumorigenesis of breast cancer MDA‑MB‑231 cells. Mol Med Rep. 2020;22:43–50. 32377708 10.3892/mmr.2020.11122 PMC7248526

[17] Lee J, Sohn EJ, Yoon SW, Kim CG, Lee S, Kim JY, et al. Anti-metastatic effect of dehydrocorydaline on H1299 non-small cell lung carcinoma cells via inhibition of matrix metalloproteinases and B cell lymphoma 2. Phytother Res. 2017;31:441–8. 28144994 10.1002/ptr.5766

[18] Kurahashi K, Fujiwara M. Adrenergic neuron blocking action of dehydrocorydaline isolated from *Corydalis bulbosa*. Can J Physiol Pharmacol. 1976;54:287–93. 953859 10.1139/y76-042

[19] Wen B, Dang YY, Wu SH, Huang YM, Ma KY, Xu YM, et al. Antiatherosclerotic effect of dehydrocorydaline on ApoE^-/-^ mice: inhibition of macrophage inflammation. Acta Pharmacol Sin. 2022;43:1408–18. 34552216 10.1038/s41401-021-00769-3 PMC9160055

[20] Sasaki T, Kuzuya M, Nakamura K, Cheng XW, Shibata T, Sato K, et al. A simple method of plaque rupture induction in apolipoprotein E-deficient mice. Arterioscler Thromb Vasc Biol. 2006;26:1304–9. 16574894 10.1161/01.ATV.0000219687.71607.f7

[21] Chi J, Meng L, Pan S, Lin H, Zhai X, Liu L, et al. Primary culture of rat aortic vascular smooth muscle cells: a new method. Med Sci Monit. 2017;23:4014–20. 28822209 10.12659/MSM.902816 PMC5572779

[22] Tie L, Xiao H, Wu DL, Yang Y, Wang P. A brief guide to good practices in pharmacological experiments: Western blotting. Acta Pharmacol Sin. 2021;42:1015–7. 33087837 10.1038/s41401-020-00539-7 PMC8209196

[23] Vengrenyuk Y, Nishi H, Long X, Ouimet M, Savji N, Martinez FO, et al. Cholesterol loading reprograms the microRNA-143/145-myocardin axis to convert aortic smooth muscle cells to a dysfunctional macrophage-like phenotype. Arterioscler Thromb Vasc Biol. 2015;35:535–46. 25573853 10.1161/ATVBAHA.114.304029 PMC4344402

[24] Sano H, Sudo T, Yokode M, Murayama T, Kataoka H, Takakura N, et al. Functional blockade of platelet-derived growth factor receptor-beta but not of receptor-alpha prevents vascular smooth muscle cell accumulation in fibrous cap lesions in apolipoprotein E-deficient mice. Circulation. 2001;103:2955–60. 11413086 10.1161/01.cir.103.24.2955

[25] Chen Y, Wang L, Huang ZS, Feng JX, Li SX, Du ZJ, et al. Cytoskeletal protein SPTA1 mediating the decrease in erectile function induced by high-fat diet via Hippo signaling pathway. Andrology. 2023;11:591–610. 36374586 10.1111/andr.13338

[26] Owens GK, Kumar MS, Wamhoff BR. Molecular regulation of vascular smooth muscle cell differentiation in development and disease. Physiol Rev. 2004;84:767–801. 15269336 10.1152/physrev.00041.2003

[27] Zhang F, Guo X, Xia Y, Mao L. An update on the phenotypic switching of vascular smooth muscle cells in the pathogenesis of atherosclerosis. Cell Mol Life Sci. 2021;79:6. 34936041 10.1007/s00018-021-04079-z PMC11072026

[28] Zhu L, Hao Y, Guan H, Cui C, Tian S, Yang D, et al. Effect of sinomenine on vascular smooth muscle cell dedifferentiation and neointima formation after vascular injury in mice. Mol Cell Biochem. 2013;373:53–62. 23065380 10.1007/s11010-012-1474-9

[29] Muller DW, Ellis SG, Topol EJ. Colchicine and antineoplastic therapy for the prevention of restenosis after percutaneous coronary interventions. J Am Coll Cardiol. 1991;17:126b–131b. 2016470 10.1016/0735-1097(91)90948-9

[30] Li Q, Wo D, Huang Y, Yu N, Zeng J, Chen H, et al. Alkaloids from Nelumbinis Plumula (AFNP) ameliorate aortic remodeling via RhoA/ROCK pathway. Biomed Pharmacother. 2019;112:108651. 30784931 10.1016/j.biopha.2019.108651

[31] Lee S, Lim HJ, Park HY, Lee KS, Park JH, Jang Y. Berberine inhibits rat vascular smooth muscle cell proliferation and migration in vitro and improves neointima formation after balloon injury in vivo. Berberine improves neointima formation in a rat model. Atherosclerosis. 2006;186:29–37. 16098530 10.1016/j.atherosclerosis.2005.06.048

[32] Wang M, Wu Y, Yu Y, Fu Y, Yan H, Wang X, et al. Rutaecarpine prevented ox-LDL-induced VSMCs dysfunction through inhibiting overexpression of connexin 43. Eur J Pharmacol. 2019;853:84–92. 30880182 10.1016/j.ejphar.2019.03.028

[33] Johnson C, Galis ZS. Matrix metalloproteinase-2 and -9 differentially regulate smooth muscle cell migration and cell-mediated collagen organization. Arterioscler Thromb Vasc Biol. 2004;24:54–60. 14551157 10.1161/01.ATV.0000100402.69997.C3

[34] Marchesi VT, Steers E Jr. Selective solubilization of a protein component of the red cell membrane. Science. 1968;159:203–4. 5634911 10.1126/science.159.3811.203

[35] Polonikov AV, Ushachev DV, Ivanov VP, Churnosov MI, Freidin MB, Ataman AV, et al. Altered erythrocyte membrane protein composition mirrors pleiotropic effects of hypertension susceptibility genes and disease pathogenesis. J Hypertens. 2015;33:2265–77. 26335431 10.1097/HJH.0000000000000699

[36] Fletcher GC, Elbediwy A, Khanal I, Ribeiro PS, Tapon N, Thompson BJ. The Spectrin cytoskeleton regulates the Hippo signalling pathway. EMBO J. 2015;34:940–54. 25712476 10.15252/embj.201489642 PMC4388601

[37] Wong KK, Li W, An Y, Duan Y, Li Z, Kang Y, et al. β-Spectrin regulates the hippo signaling pathway and modulates the basal actin network. J Biol Chem. 2015;290:6397–407. 25589787 10.1074/jbc.M114.629493 PMC4358275

