# Supplementary figures and images for: Dehydrocorydaline maintains the vascular smooth muscle cell contractile phenotype by upregulating Spta1
Source: Acta Pharmacol Sin. 2025 Jan 20;46(5):1303–16. doi: 10.1038/s41401-024-01464-9 (PMC12032006; doi:10.1038/s41401-024-01464-9)

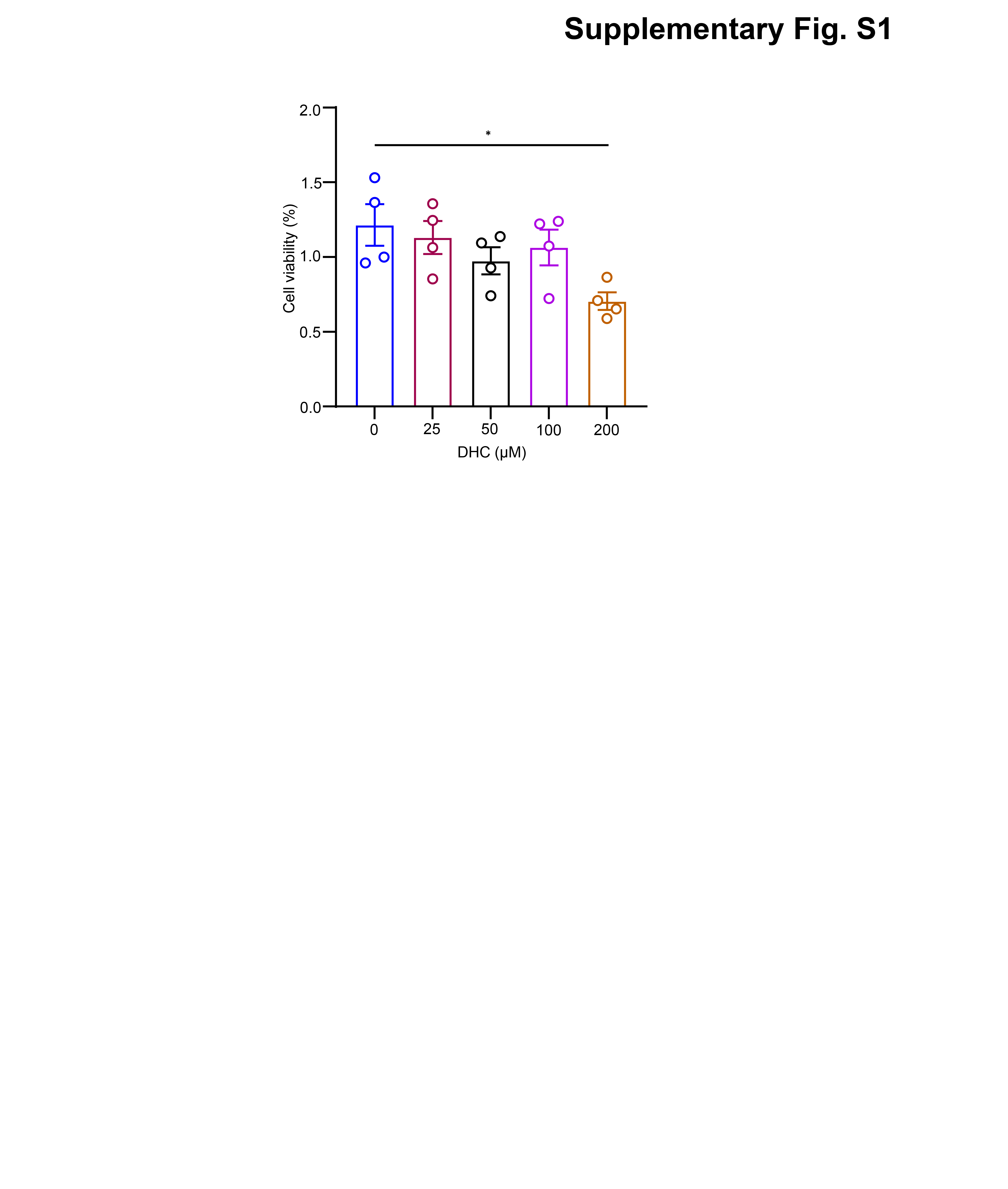

Supplement: Supplementary file 1 — Supplementary Figure S1 [file 41401_2024_1464_MOESM1_ESM.tif]

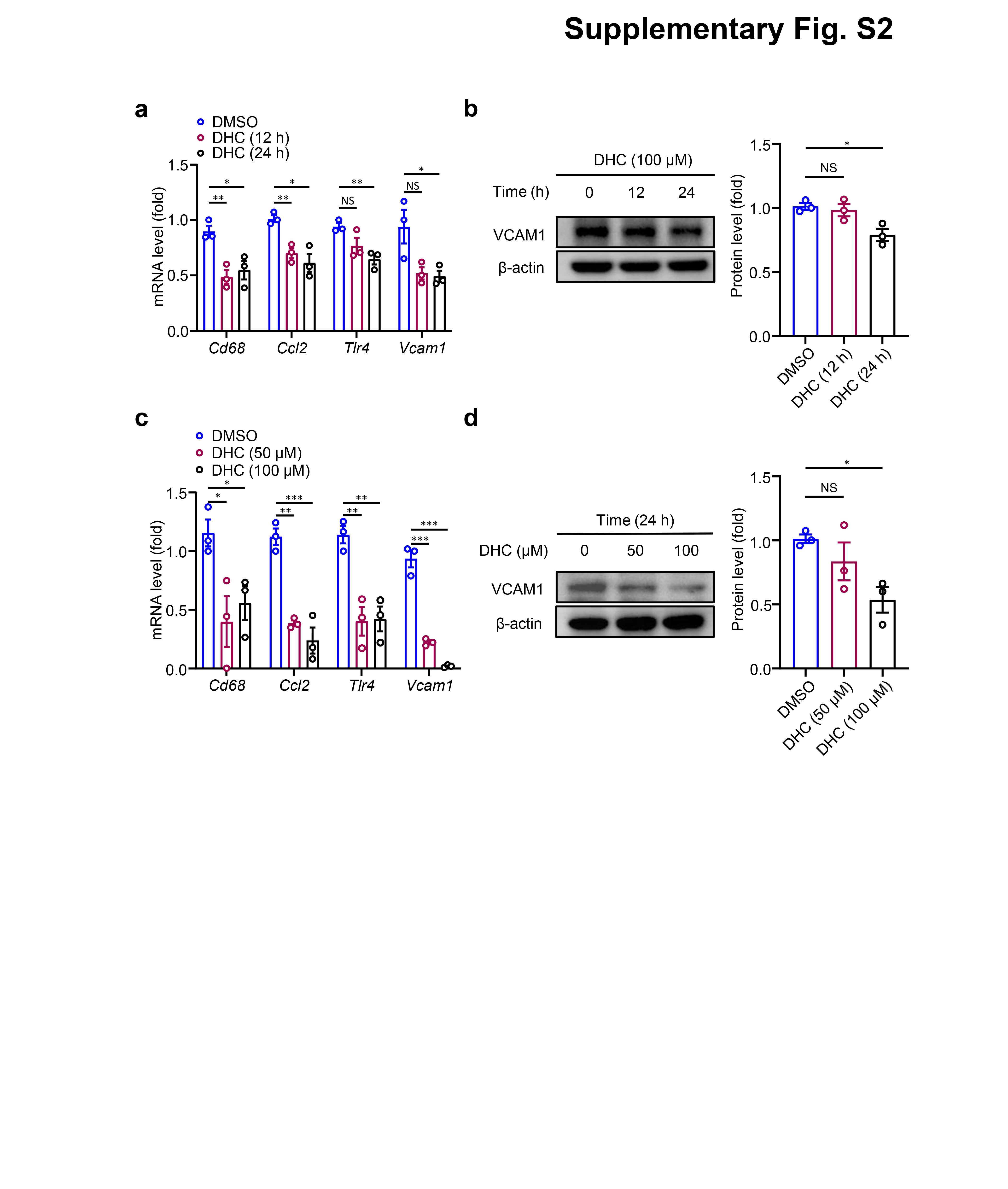

Supplement: Supplementary file 2 — Supplementary Figure S2 [file 41401_2024_1464_MOESM2_ESM.tif]

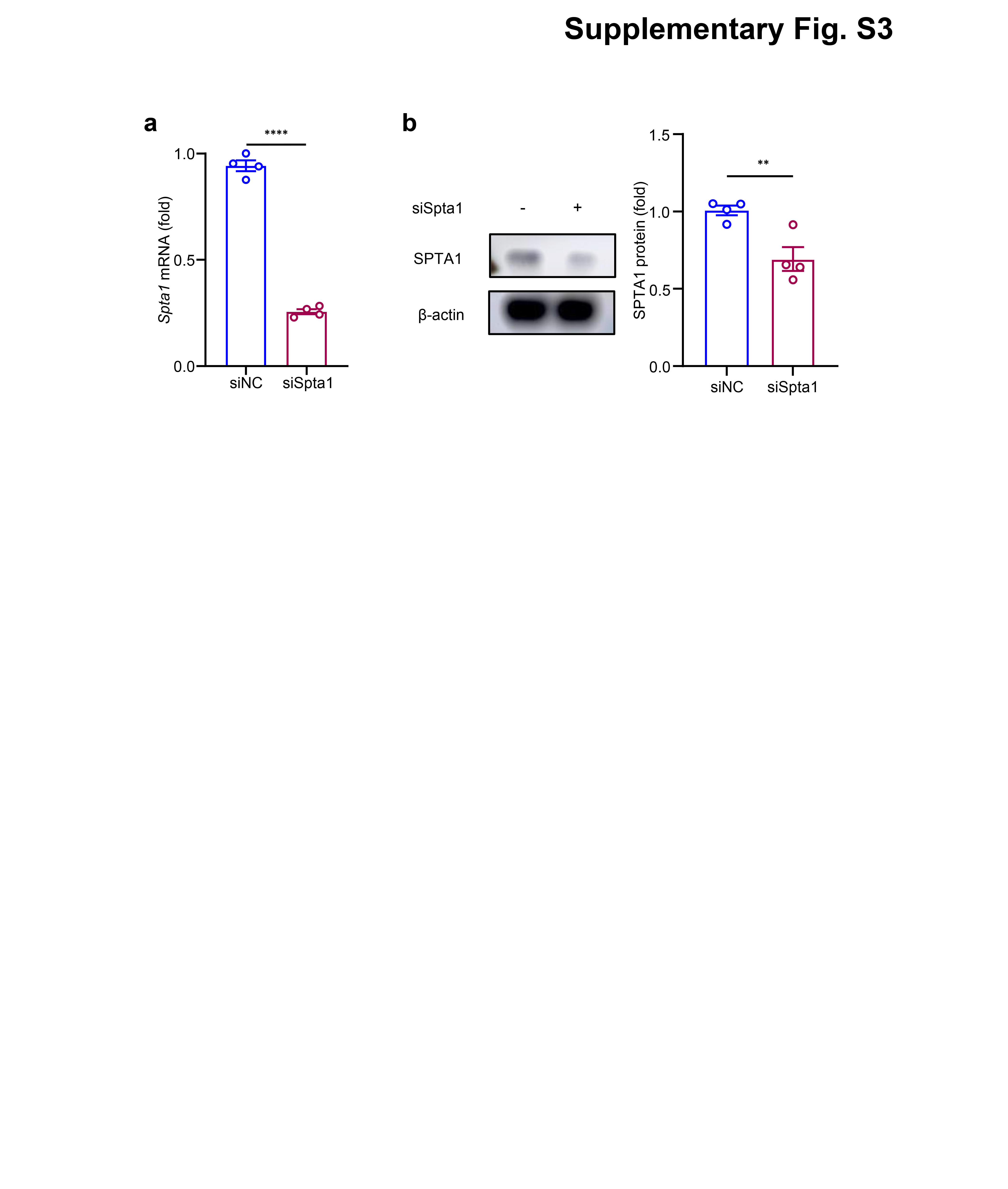

Supplement: Supplementary file 3 — Supplementary Figure S3 [file 41401_2024_1464_MOESM3_ESM.tif]

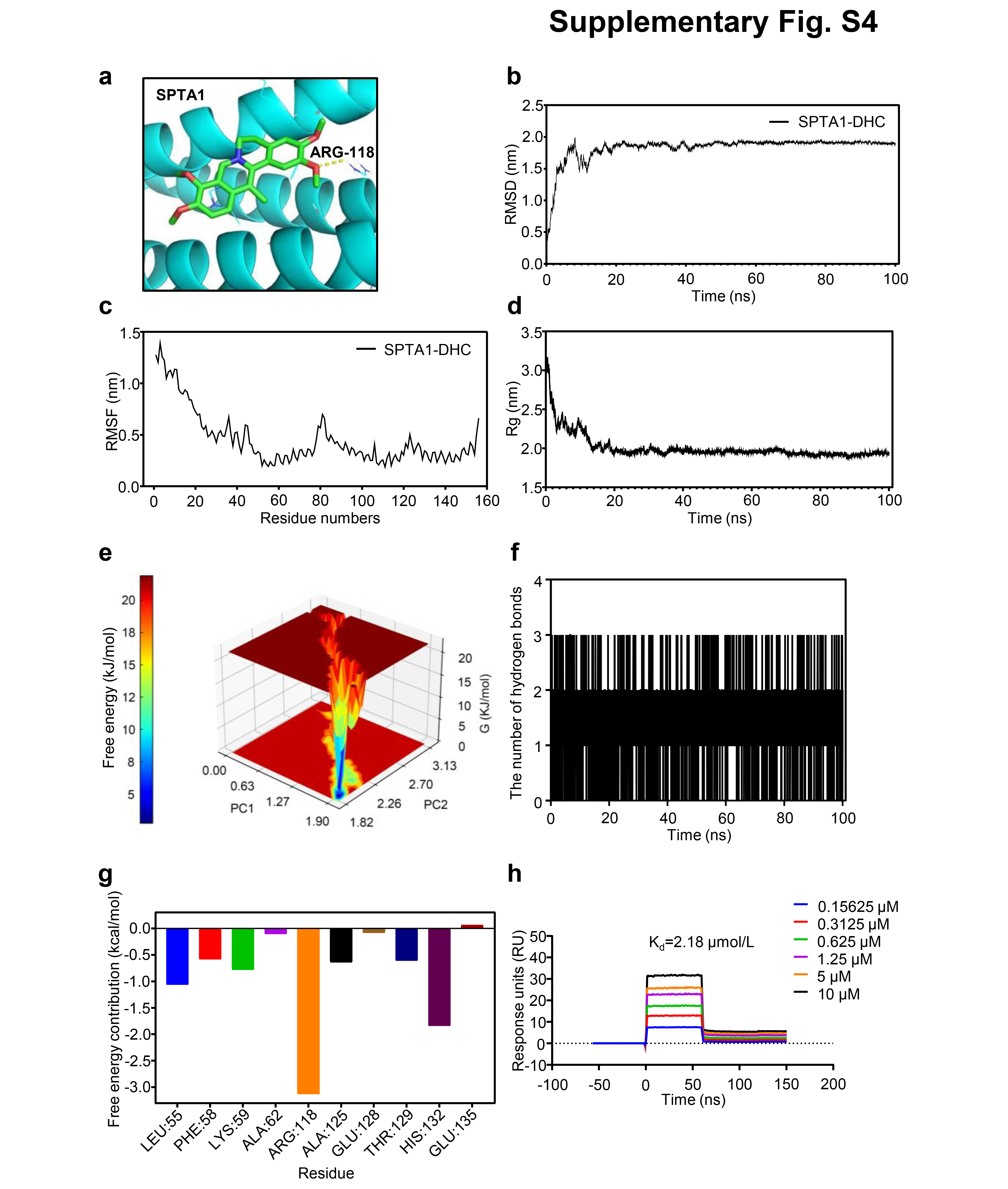

Supplement: Supplementary file 4 — Supplementary Figure S4 [file 41401_2024_1464_MOESM4_ESM.tif]
