# Supplementary material for: Dehydrocorydaline maintains the vascular smooth muscle cell contractile phenotype by upregulating Spta1
Source: Acta Pharmacol Sin. 2025 Jan 20;46(5):1303–16. doi: 10.1038/s41401-024-01464-9 (PMC12032006; doi:10.1038/s41401-024-01464-9)

Figure 2b

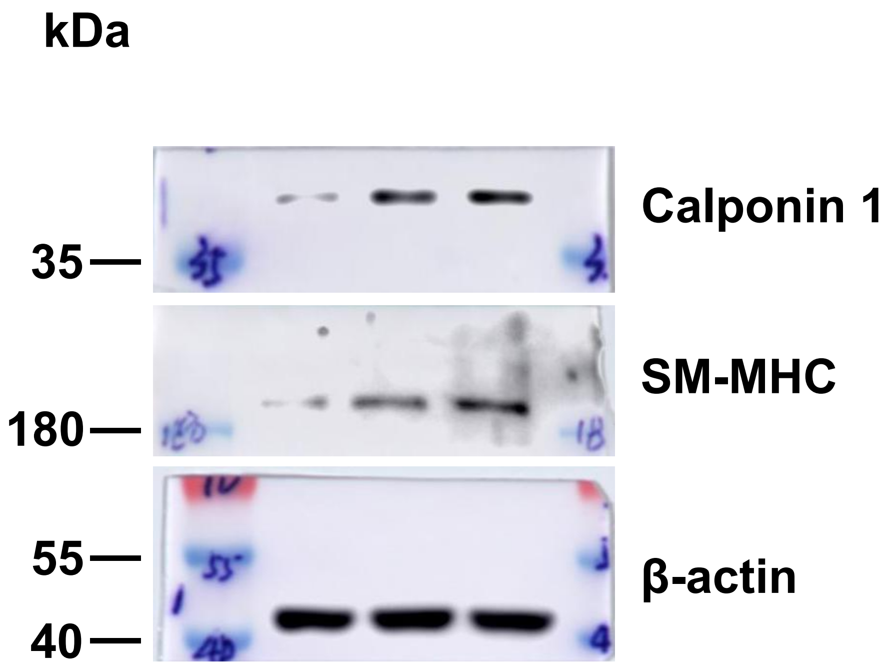

Figure 2d

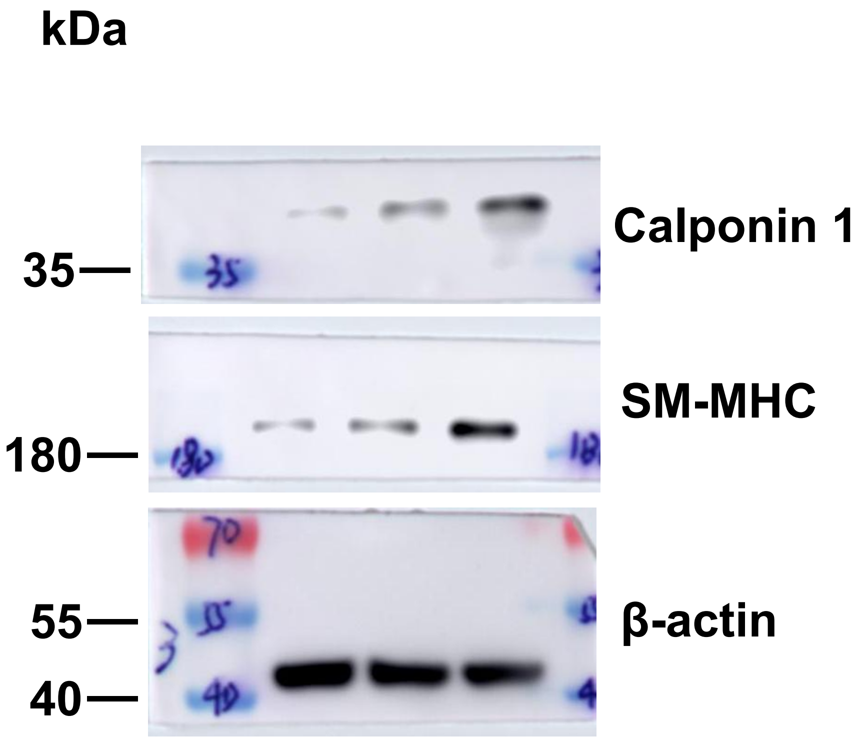

Figure 3b

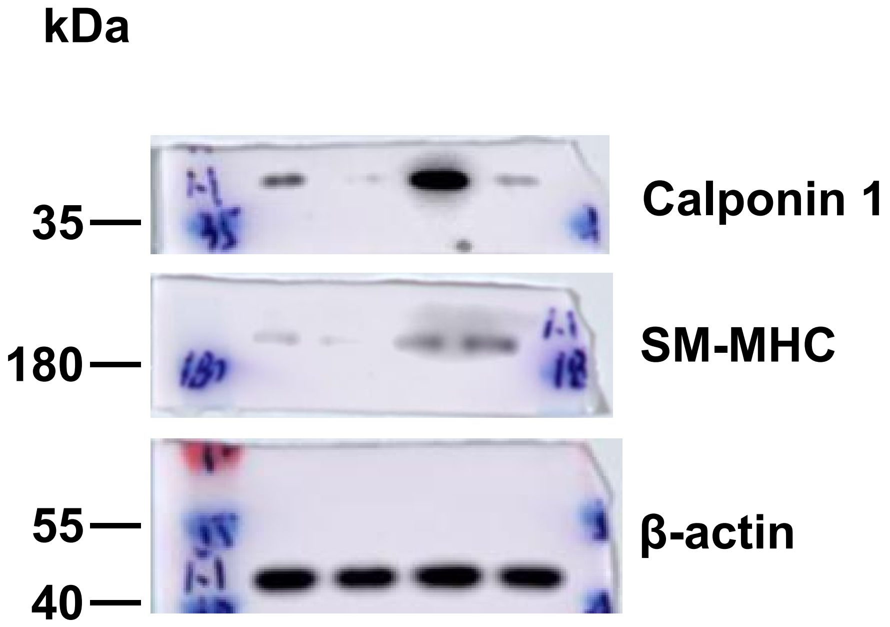

Figure 7c

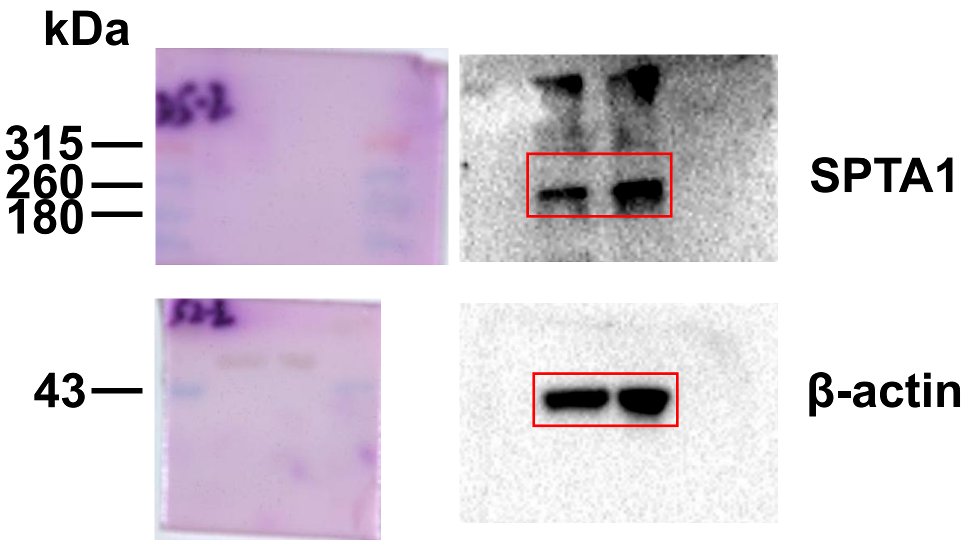

Figure 7e

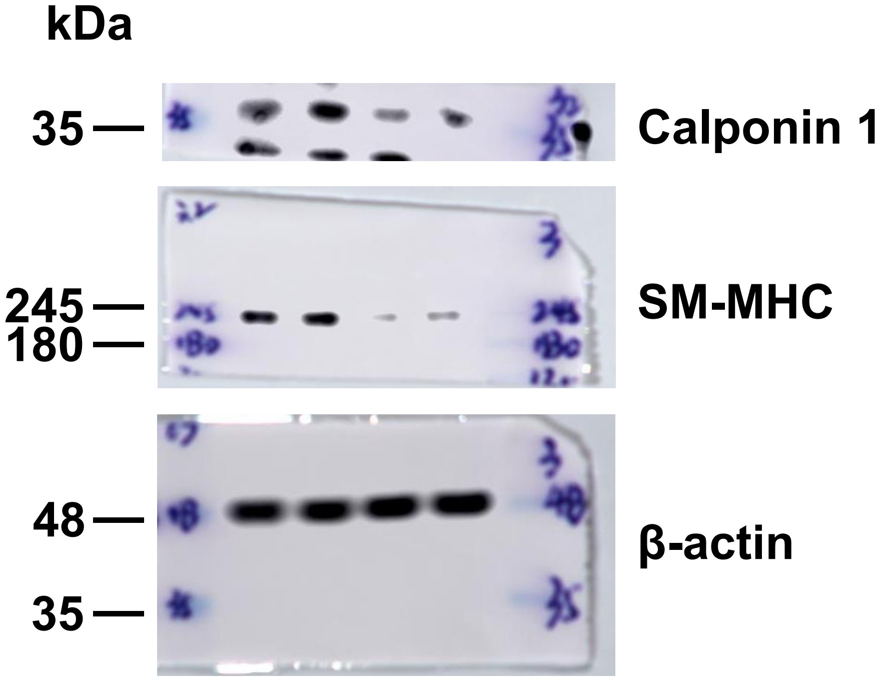

Supplementary figure S2b

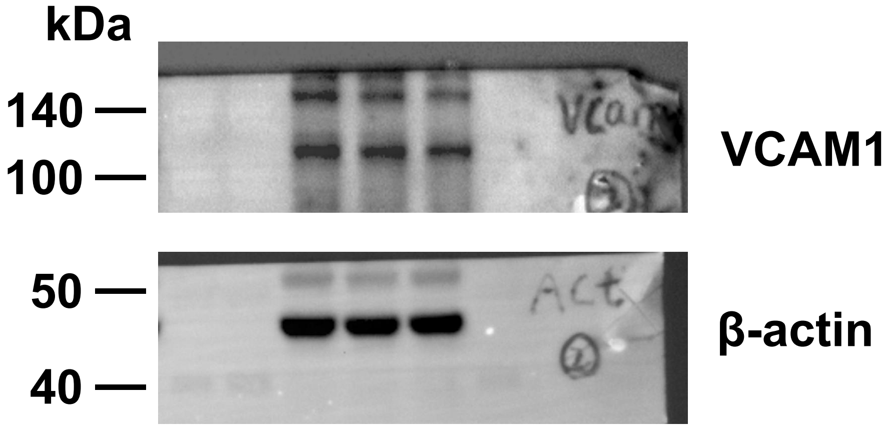

Supplementary figure S2d

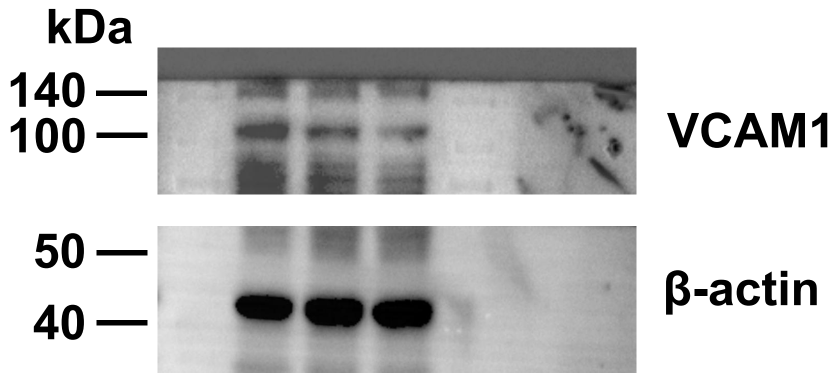

Supplementary figure S3b

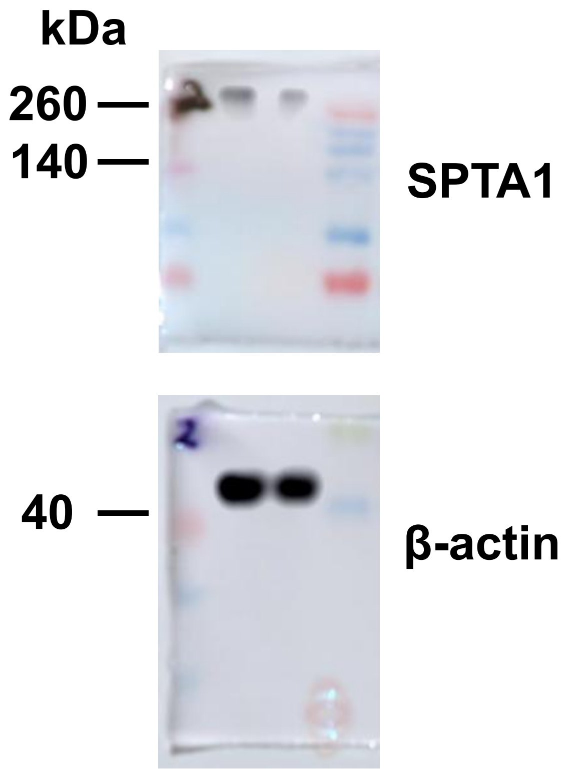

Supplement: Supplementary file 5 — Uncropped blots [file 41401_2024_1464_MOESM5_ESM.pdf]
