# Supplementary material for: Dehydrocorydaline maintains the vascular smooth muscle cell contractile phenotype by upregulating Spta1
Source: Acta Pharmacol Sin. 2025 Jan 20;46(5):1303–16. doi: 10.1038/s41401-024-01464-9 (PMC12032006; doi:10.1038/s41401-024-01464-9)
Supplement: Supplementary file 6 — Supplementary figure legend [file 41401_2024_1464_MOESM6_ESM.docx]

**Supplementary Fig. S1. The cytotoxicity of DHC on rat VSMCs was determined using a CCK-8 assay.** Data are presented as the mean ± SEM. **P* < 0.05, analyzed using unpaired *t*-tests.

**Supplementary Fig. S2. DHC inhibits the expression of inflammatory factors in VSMCs.** (**a** and **b**) Rat VSMCs were treated with 100 μM DHC for 0, 12, and 24 h. (**a**) The mRNA levels of *Cd68*, CC chemokine ligand 2 (*Ccl2*), Toll-like receptor 4 (*Tlr4*), and vascular cell adhesion molecule 1 (*Vcam1*) were detected by RT-qPCR (*n* = 3). (**b**) The protein level of VCAM1 was detected by western blotting (*n* = 3). (**c** and **d**) Rat VSMCs were treated with 0, 50, and 100 μM DHC for 24 h. (**c**) The mRNA levels of *Cd68*, *Ccl2*, *Tlr4*, and *Vcam1* were detected by RT-qPCR (*n* = 3). (**d**) The protein level of VCAM1 was detected by western blotting (*n* = 3). Data are presented as the mean ± SEM. NS indicates not significant, **P* < 0.05, ***P* < 0.01, and ****P* < 0.001, analyzed using unpaired *t*-tests.

**Supplementary Fig. S3. Verification of siSpta1 knockdown efficiency.** (**a** and **b**) Rat VSMCs were transfected with siSpta1 for 24 h. The mRNA and protein levels of SPTA1 were determined using RT-qPCR (**a**) and western blotting (**b**) (*n* = 4). Data are presented as the mean ± SEM. ***P* < 0.01, *****P* < 0.0001, analyzed using unpaired *t*-tests.

**Supplementary Fig. S4. Analysis of the interaction between SPTA1 and DHC.** (**a**) Molecular docking analysis of SPTA1 and DHC. (**b**) The root mean square deviation plot of the SPTA1-DHC complex. (**c**) The mean square fluctuation plot of the SPTA1-DHC complex. (**d**) The radius of gyration plot of the SPTA1-DHC complex. (**e**) The Gibbs energy landscape of the SPTA1-DHC complex. (**f**) The number of hydrogen bonds in the SPTA1-DHC complex. (**g**) The free energy contribution of the SPTA1-DHC complex. (**h**) Surface plasmon resonance (SPR) analysis of DHC binding to SPTA1 (K_d_ = 2.18 μM).
